# Severe Pertussis Infection With Hyperleukocytosis in a 10-Month-Old Unvaccinated Amish Female: A Case Report

**DOI:** 10.7759/cureus.26885

**Published:** 2022-07-15

**Authors:** Stephen Long, Robert B Lowe

**Affiliations:** 1 Internal Medicine & Pediatrics, Geisinger Commonwealth School of Medicine, Scranton, USA; 2 Hospital Medicine, Geisinger Health System, Danville, USA

**Keywords:** bordetella pertussis, pediatric pulmonary hypertension, leukoreduction, unvaccinated, hyperleukocytosis

## Abstract

*Bordetella* *pertussis (B. pertussis) *commonly infects individuals of all ages. However, pertussis, the disease caused by *B. pertussis* infection, is most severe in young infants. Severe pertussis, defined by the presence of refractory hypoxemia, pneumonia, cardiogenic shock, and hyperleukocytosis, is associated with significant morbidity and mortality. Both hyperleukocytosis and pulmonary hypertension have been found to be predictive of mortality in young infants. Leukoreductive strategies such as leukapheresis and exchange transfusion have been employed to treat these complications. Pulmonary hypertension is thought to be a result of aggregation of white blood cells in pulmonary vasculature; however, studies have suggested that the mechanism of pulmonary hypertension is multifactorial. We report a case of a 10-month-old unvaccinated Amish female with pertussis complicated by an initial hyperleukocytosis of 204,900 10^3^/uL successfully treated with leukapheresis in our pediatric intensive care unit. This infant never showed signs of pulmonary hypertension, which is often associated with hyperleukocytosis in severe or fatal cases of pertussis in infants and neonates. To our knowledge, this is the most significant degree of hyperleukocytosis reported in pertussis. The findings in this case support the clinical utility of leukoreductive therapy in severe pertussis and provide some evidence that the mechanism of pulmonary hypertension in these patients is multifactorial.

## Introduction

*Bordetella pertussis (B. pertussis)* infection is one of the most poorly controlled vaccine-preventable diseases within the United States, with outbreaks and incidence increasing over the past two decades [1]. Pertussis in neonates and young infants is frequently associated with hyperleukocytosis, pulmonary hypertension, and hypoxemia [2-7]. Hyperleukocytosis, with peak white blood cell (WBC) counts often surpassing 100,000, is a frequently reported phenomenon of pertussis, with many retrospective observational studies linking its presence to adverse outcomes and mortality [2,4,8-17]. Hyperleukocytosis typically occurs in cases of severe pertussis, which is defined by pneumonia with refractory hypoxemia, cardiogenic shock, and severe hyperleukocytosis [9]. The development of pulmonary hypertension in these patients is thought to be a result of leukocyte-rich thrombi becoming lodged in the pulmonary arterial system [16,18]. Hyperhydration, exchange transfusion, and leukapheresis have been employed successfully to prevent and treat pulmonary hypertension in cases of pertussis complicated by hyperleukocytosis [8-14]. We report a case of a 10-month-old unvaccinated Amish female with severe *B. pertussis* infection complicated by an initial WBC of 204.9 10^3^/uL treated successfully with hyperhydration and leukoreduction via leukapheresis.

## Case presentation

A 10-month-old, previously healthy Amish female presented to a local emergency department for evaluation of one week of worsening cough, several episodes of post-tussive emesis, reduced urine output, and increased work of breathing. This child was born at home and received no vaccinations. On examination, she was found to be febrile at 38.3 °C with a respiratory rate of 38 breaths/minute, oxygen saturation of 88% on room air, tachycardic with a heart rate of 180 BPM, and hypertensive with a blood pressure of 130/90. Chest examination demonstrated clear lung sounds bilaterally but accessory muscle use. Complete blood count showed marked hyperleukocytosis with a white blood cell count (WBC) of 204.90 10^3^/uL with 44% neutrophils and 46% lymphocytes and a platelet count of 780.0 10^3^/uL (Table 1). Peripheral saturation improved with supplemental oxygen via nasal cannula. She received intravenous azithromycin (10 mg/kg) and ceftriaxone (50 mg/kg) and was transferred to the pediatric intensive care unit (PICU) for further management. A multiplex polymerase chain reaction (PCR) test for common respiratory pathogens was positive for *Bordetella pertussis* only (Table 2). Flow cytometry of a blood sample showed no evidence of a neoplastic process. A bone marrow biopsy confirmed the flow cytometry findings. 

**Table 1:** Complete blood count

	Reference range	Value
White blood cells (WBC)	5.00 - 17.50 K/uL	204.90
Red blood cells (RBC)	3.70 - 5.30 M/uL	4.5
Hemoglobin	10.5 - 13.5 g/dL	10.5
Hematocrit	33.0 - 39.0 %	34.5
Mean corpuscular volume (MCV)	70.0 - 86.0 fL	76.7
Mean corpuscular hemoglobin (MCH)	23.0 - 31.0 pg	23.3
Mean corpuscular hemoglobin concentration ( MCHC)	32.0 - 36.0 g/dL	30.4
Red cell distribution width (RDW)	11.5 - 15.5 %	14.8
Platelets	140 - 400 K/uL	780
Mean platelet volume (MPV)	6.6 - 11.1 fL	9.5
Neutrophils %	27 - 51 %	44
Lymphocytes %	16 - 46 %	46
Monocytes %	1 - 11 %	5
Eosinophils %	0 - 6 %	1

**Table 2:** Polymerase chain reaction for respiratory pathogens NEG = Negative test

	Reference range	Value
Adenovirus	NEG	NEGATIVE
Coronavirus 229E	NEG	NEGATIVE
Coronavirus HKU1	NEG	NEGATIVE
Coronavirus NL63	NEG	NEGATIVE
Coronavirus OC43	NEG	NEGATIVE
Human metapneumovirus	NEG	NEGATIVE
Rhinovirus/enterovirus	NEG	NEGATIVE
Influenza A virus	NEG	NEGATIVE
Influenza B virus	NEG	NEGATIVE
Parainfluenza virus 1	NEG	NEGATIVE
Parainfluenza virus 2	NEG	NEGATIVE
Parainfluenza virus 3	NEG	NEGATIVE
Parainfluenza virus 4	NEG	NEGATIVE
Respiratory syncytial virus	NEG	NEGATIVE
Bordetella pertussis	NEG	POSITIVE
Chlamydophila pneumoniae	NEG	NEGATIVE
Mycoplasma pneumoniae	NEG	NEGATIVE

Aggressive intravenous fluid hydration was initiated with 40 ml/kg of normal saline bolus followed by D5 normal saline at maintenance dose. A transesophageal echocardiogram did not reveal any evidence of pulmonary hypertension on day one of hospitalization. Due to the high risk of developing hyperviscosity-related complications and small vessel occlusion, the decision was made to proceed with double-volume leukoreduction via apheresis with a goal WBC of less than 50.0 10^3^/uL. She underwent the first round of leukapheresis on day one of hospitalization, which resulted in a reduction of her WBC to 98.45 10^3^/uL with 25% lymphocytes and 64% neutrophils. Unfortunately, this was complicated by worsening interstitial edema thought to be secondary to the rapid change in oncotic pressure produced by leukapheresis. Bilevel positive airway pressure (BiPAP) ventilation was initiated. A second leukoreduction was carried out on day two of hospitalization without significant improvement. A third leukoreduction targeting granulocytes was conducted on the hospital day three, and her WBC was reduced to 50.62 10^3^/uL before rebounding to 73.8 10^3^/uL twelve hours later. She did not tolerate attempts to wean respiratory support; therefore, the decision was made to proceed with single-volume apheresis to reduce the circulating volume of pertussis toxin (PT) on hospital day four. This resulted in the improvement of her respiratory status allowing supplemental oxygen wean. She continued to make slow improvements despite complications such as bacterial pneumonia on day eight of her hospitalization, which was successfully treated with intravenous ceftriaxone. She was ultimately discharged from the hospital without supplemental oxygen after 21 days. Her WBC count at discharge was 24.9 10^3^/uL with 26% lymphocytes and 58% neutrophils.

## Discussion

Pertussis remains a common infectious disease in individuals of all ages worldwide and can produce a wide spectrum of clinical manifestations, ranging from upper respiratory symptoms to hypoxia, hypotension, and shock. The severity of disease in young infants is influenced by several factors, including maternal vaccination during pregnancy, infant age and weight, vaccination status, and breastfeeding [4]. The most significant risk factor for neonatal infection appears to be a large family size [4]. This 10-month-old, unvaccinated female is a member of a self-containing Amish community. Although the Amish beliefs do not universally reject all vaccinations, rates of vaccination in these communities are significantly lower than national averages. Children in similar communities have been found to be at increased risk of hospitalization due to vaccine-preventable illnesses.

Hyperleukocytosis with lymphocytic predominance is a well-documented occurrence in severe pertussis, with cases reported throughout the 1900s until recent years [8-14]. This phenomenon occurs more frequently in young infants than in older children and adults [5]. Several retrospective studies have established hyperleukocytosis as an indicator of increased mortality in young infants and an independent predictor of mortality in all patients [2,4,15-17]. One prospective cohort study of 127 patients found that infants with an initial WBC greater than 50.0 10^3^/uL had a relative risk of death of 9.8 [2].

Hyperleukocytosis in pertussis is thought to be caused by pertussis toxin (PT), which functions as an inhibitor of G-protein coupled receptor (GPCR) signaling [19]. In animal models, PT has been shown to reduce the expression of several cell-surface markers, such as L-selectin, which are needed for lymphocyte extravasation into lymphoid tissue [9]. This results in an increase in the number of circulating lymphocytes within the vasculature. 

Infants with higher peak WBC levels are at an increased risk of developing pulmonary hypertension which is associated with increased mortality [2]. Hyperleukocytosis leading to intravascular leukocyte-rich thrombi in the pulmonary vasculature is one proposed mechanism [7]. While this theory is supported by several post-mortem histologic analyses of pulmonary tissue from infant victims of pertussis, other similar studies from post-mortem tissue analysis have found the presence of thrombi to be inconsistent, suggesting a multifactorial mechanism [7, 16,18].

Hyperleukocytosis and pulmonary hypertension appear to be most common in infants <120 days old, suggesting expression and function of GPCRs, such as PT, may change throughout development [19]. For example, the angiotensin-II receptor AT2 has been shown to be highly expressed in fetal tissue before being balanced by AT1 receptors in childhood [20]. The AT2 receptor helps regulate smooth muscle cell production in vasculature. Inhibition of AT2 by pertussis toxin could result in unopposed smooth muscle cell proliferation leading to pulmonary arterial thickening, vasoconstriction, and pulmonary hypertension [19]. A histopathologic study by Winter and Harriman demonstrated medial thickening of pulmonary arteries in four out of five infants after fatal pertussis complicated by pulmonary hypertension [14]

Due to the well-established relationship between hyperleukocytosis and mortality, several strategies have been employed to reduce high WBC counts. Case reports have described the successful use of leukoreduction by either exchange transfusion or leukapheresis. One study provided clear indication that leukoreduction may be associated with ICU survival [18]. There is currently no evidence favoring the use of leukapheresis over exchange transfusion or vice versa. This clinical decision depends in large part on resource and staff availability.

To our knowledge, this is the highest degree of hyperleukocytosis secondary to pertussis reported in the literature. Additionally, this child did not develop echocardiographic signs of pulmonary hypertension despite her marked leukocytosis of greater than >200.0 10^3^/uL. Given the studies suggesting that higher WBC counts are correlated with, and may even be causative of, the development of pulmonary hypertension, the lack of this finding in this case suggests that hyperleukocytosis is one aspect of a multifactorial mechanism. Moreover, most cases of hyperleukocytosis occurred in infants less than 120 days old, while this infant was 10 months old at the time of admission. This may indicate that older infants are less susceptible to the development of pulmonary hypertension [2]. While there are no clear guidelines on when in the clinical course to perform leukoreductive therapy in severe pertussis, this infant underwent consecutive rounds of leukapheresis on the first three days of hospitalization. Prompt initiation of leukoreductive therapy in this child may have reduced the risk of developing pulmonary hypertension later in the clinical course.

## Conclusions

We report a case of dramatic hyperleukocytosis in a 10-month-old unvaccinated female successfully treated with leukapheresis. This case underscores the need for improved vaccination rates among target communities and provides some insight into the complex interaction between hyperleukocytosis and pulmonary hypertension. Several studies have proposed guidelines for the use of leukoreduction in cases of severe pertussis; however, this evidence is still evolving, and no formal, society-backed recommendations exist. Even more scarce is the data available for older infants with extreme hyperleukocytosis. We believe the outcome of this case supports the viability of leukoreduction via leukapheresis in severe pertussis. This treatment likely provides benefits by reducing both the number of circulating leukocytes and the amount of circulating pertussis toxin and may help prevent or decrease the risk for developing pulmonary hypertension leading to decreased mortality. Further studies are needed to gain insight into the preferred timing, method, and duration of leukoreductive therapy in pertussis. 
